# Exercício Físico em Pacientes Cardiopatas e na População em Tempos de Coronavírus

**DOI:** 10.36660/abc.20200281

**Published:** 2020-05-22

**Authors:** Ricardo Stein

**Affiliations:** 1 Universidade Federal do Rio Grande do Sul Porto AlegreRS Brasil Universidade Federal do Rio Grande do Sul, Porto Alegre, RS – Brasil

**Keywords:** Coronavirus, COVID-19, Exercício, Atividade Física, Estilo de Vida, Sedentarismo, Doenças Cardiovasculares, Fatores de Risco, Dieta Saudável, Residência

Começo este texto sem falar de exercício. Quero frisar que portadores de doenças crônicas como hipertensão, insuficiência cardíaca, diferentes tipos de miocardiopatias e algumas formas de arritmias estão sob risco aumentado para desfechos fatais após contraírem o SARS-COV-2 e desenvolverem a infecção denominada COVID-19. Da mesma forma, quem realizou revascularização mecânica ou cirúrgica, portadores de próteses valvares (biológicas ou metálicas), de dispositivos como marca-passo ou desfibrilador implantado (entre outros), assim como aqueles com algumas formas de cardiopatias congênitas, também fazem parte de um grupo de maior risco.^1^

**Ponto número um:** Os pacientes acima são definitivamente de maior risco e eles fazem parte do grupo de risco em qualquer idade.^*^

^*^Observação: Assim como os cardiopatas, os idosos, os imunossuprimidos e os portadores de algumas doenças crônicas (como o diabetes e as doenças pulmonares), também têm risco aumentado ao serem infectados.

- Mas a pergunta que não quer calar: E o exercício nos cardiopatas e na população geral, como devemos proceder? É consenso que a prática do exercício físico regular deve continuar, mesmo no isolamento em casa, sendo essa uma posição clara entre cardiologistas, médicos do exercício e demais especialistas. Claro que por um lado poderá parecer mais difícil, mas por outro, a maior disponibilidade de tempo para muitos acabará com a desculpa do “não tenho tempo”. A semana continua tendo 168 horas. Será que não dá para dedicar cinco períodos de meia hora, pelo menos, para fazer algum tipo de exercício dentro de casa?

**Ponto número dois:** Exercitar-se dentro de sua casa é indicado pelos dois motivos seguintes: a) respeita o critério principal de cuidados contra a infecção pelo SARS-COV-2 que causa a COVID-19; b) faz bem para saúde.

- No entanto, surge outra pergunta: Se me sinto bem, por que não posso me exercitar na rua, nas praças ou nas academias dos condomínios? Se pensarmos de forma individual, sair à rua ou correr na esteira do condomínio não parece ser problemático. Agora, o pensamento social e epidemiológico vai na direção de que a chance de aglomeração aumenta e isso vai de encontro ao que é preconizado pelos especialistas. Imaginem se todos decidissem ir ao calçadão situado na beira de uma cidade que tenha praia ou a um parque dar uma corridinha ao mesmo tempo… Seria a glória… para o vírus!!!

**Ponto número três:** Na relação risco-benefício os especialistas recomendam que não se saia de casa para fazer exercício neste momento pandêmico.

- Outra pergunta que me parece importante: E se eu estiver com algum sinal e/ou sintoma, devo me exercitar? Não!!! O exercício não deve ser realizado em vigência de sinais e/ou sintomas, seja nesta ou em qualquer outra situação de infecção. O melhor a se fazer é repousar até a plena recuperação.

**Ponto número quatro:** Se sintomático, não fazer exercício, nem mesmo dentro de casa. Após recuperar-se, a volta a prática passa a ser indicada.

- Outro ponto relevante diz respeito à história prévia de exercício. Que bom que todos fossem fisicamente ativos e a inatividade fosse algo muito pontual. Alguns pensam que por serem ativos ou atléticos estão imunes ao contagio pelo SARS-COV-2. Nada disso!!! Atletas de alta performance já contraíram COVID-19 e convalesceram ou convalescem, alguns até tendo sido internados.

**Ponto número cinco:** Indivíduos ativos ou atletas devem tomar os mesmos cuidados do resto da população, estando sob risco de contrair a doença como qualquer outro ser humano.

- Muitos de nós tivemos uma infância rica no que diz respeito à atividade física. Brincamos de bambolê, cabra cega, sapata (amarelinha) e de stop. Pulamos corda, brincamos de esconder, fizemos cabo de guerra, entre outras brincadeiras que ficaram na memória, que evocam um tempo sem pandemia, mas que inexoravelmente não voltará mais. Nesse cenário, por que não aproveitar o tempo com seus filhos; repetindo: aproveitar o tempo com seus filhos, algo inimaginável para muitos até dias atrás!!! Arraste-os da cama e do sofá, incentive-os a sair um pouco do WhatsApp, dos jogos e do Netflix. Vá brincar!!!

**Ponto número seis:***De volta para o passado* . Incentive seus filhos a terem um comportamento menos sedentário. O ideal, segundo a Organização Mundial de Saúde, é que o adulto dispense 150 minutos e os mais jovens se exercitem pelo menos 300 minutos por semana.^2^ Em suma, integração familiar em prol da saúde da própria família.

- Nem toda atividade física é um exercício, mas todo exercício é uma forma de atividade física. Os exemplos são inúmeros: varrer, passar, limpar e/ou aspirar a casa, jardinagem, trabalho na horta, subir e descer escadas (tomar cuidado se as escadas de um prédio estiverem sendo usadas por outros moradores ao mesmo tempo), entre outros.^2^

**Ponto número sete:** Intensificar a atividade física dentro de casa no dia a dia. Você e seus familiares terão uma vida mais ativa, saudável e útil.

- Atividade sexual é uma forma de exercício/atividade física. Para muitos a mais prazerosa dentre todas elas. Em tempos do binômio isolamento-coronavírus essa é uma preocupação generalizada. Com certeza não dispomos de dados científicos, mas a lógica nos faz pensar que o distanciamento deva prevalecer ao desejo. Que o bom senso deva ser maior do que o princípio do prazer.

**Ponto número oito:** Infelizmente, se recomenda que a atividade sexual que envolve abraços, carícias, beijos e penetração deva ser evitada (no cenário da atividade sexual ocasional, é claro). A masturbação, o uso de vibradores e de brinquedos eróticos não tem contraindicação. Se estes forem compartilhados, a indicação da lavagem com água e sabão e/ou o uso do álcool e gel 70% me parece ser indicado (dedução meramente intuitiva, pois não existem estudos testando qualquer hipótese nesse particular).

- Leitores dos Arquivos, certamente poderiam ser elencados outros vários pontos no que tange esse tema, mas me parece que no final destas poucas linhas, o que vale é o reforço de que a inatividade física é um inimigo nefasto. Seus efeitos não costumam se manifestar de forma aguda como são os provocados pelo SARS-COV-2, os quais devem ser combatidos com medidas emergenciais de saúde embasadas nas melhores evidências científicas. Por sua vez, ao afetar negativamente os sistemas cardiovascular, respiratório, metabólico e muscular, assim como o sistema imune, a inatividade física também é muito prejudicial. Em suma, os oito pontos acima foram escritos para que você reflita e pense que hoje temos de viver nos moldes impostos por essa nova ordem global, restritiva, mas não limitante.
