# Supplementary material for: Exercício Físico em Pacientes Cardiopatas e na População em Tempos de Coronavírus
Source: Arq Bras Cardiol. 2020 May 22;114(5):827–8. [Article in Portuguese] doi: 10.36660/abc.20200281 (PMC8386989; doi:10.36660/abc.20200281)
Supplement: Translation

# Translation: Physical Exercise in Patients with Heart Disease and in the General Population in Times of Coronavirus

**Authors:** Ricardo Stein

**Affiliations:** 1 Brazil Universidade Federal do Rio Grande do Sul, Porto Alegre, RS – Brazil

**Keywords:** Coronavirus, COVID-19, Exercise, Physical Activity, Life Style, Sedentarism, Cardiovascular Diseases, Risk Factors, Healthy Diet, Residence

I will begin this text without speaking about exercise. I want to emphasize that individuals with chronic diseases, such as hypertension, heart failure, different types of myocardiopathy, and some forms of arrhythmia are at an increased risk of fatal outcomes after contracting SARS-COV-2 and developing the infection denominated COVID-19. In the same manner, those who have undergone mechanical or surgical revascularization, individuals with prosthetic valves (whether biological or metallic), pacemakers, implanted defibrillators, or other devices, as well as those with some forms of congenital heart disease are also part of a higher risk group.^1^

**First point** : The patients described above are definitely at a higher risk and they are part of the risk group regardless of age.^*^

^*^Note: Like individuals with heart disease, the elderly, immunosuppressed patients, and individuals with some chronic diseases, such as diabetes and pulmonary disease, are also at an increased risk of being infected.

- This raises the question, “Regarding exercise in individuals with heart disease and in the general population, how should we proceed? There is a consensus that regular practice of physical exercise should continue, even in isolation at home; this position is clear among cardiologists, exercise physicians, and other specialists. Naturally, on one hand this may seem difficult, but, on the other, the increase in available time will do away with the excuse that “I don’t have time.” The week continues to be made up of 168 hours. Is it not possible to dedicate five half-hour periods, at least, to some type of exercise at home?

**Second point:** Exercising at home is indicated for the following two reasons: a) It follows the main criterion of care against SARS-COV-2 infection that causes COVID-19; b) it is healthy.

- This, however, brings up another question: If I feel well, why can’t I exercise in the street, in public squares, in gyms, or in condominiums? If we think in individual terms, going out or running on the condominium treadmill does not appear to be problematic. However, social and epidemiological reasoning indicates that the chance of there being a crowd increases and this goes against specialists’ recommendations. Imagine if everyone decided to go to the sidewalk next to the beach or to the park to jog at the same time… It would be wonderful… for the virus!!!

**Third point:** In the risk-benefit ratio, specialists recommend not leaving home to exercise during this pandemic moment.

- Another question seems important to me: If I have signs and/or symptoms, should I exercise? No!!! Exercise should not be practiced in the presence of signs and/or symptoms, neither in this nor in any other situation of infection. The best thing to do is rest until you have fully recovered.

**Fourth point:** If you are symptomatic, do not exercise, not even at home. After you have recovered, return to practicing as indicated.

- Another relevant point deals with previous history of exercise. It would be great if everyone were physically active and inactivity were a minor issue. Some people think that because they are active or athletic they are immune to SARS-COV-2. Not at all!!! High performance athletes have already contracted COVID-19 and recovered or are in recovery; some have even been hospitalized.

**Fifth point:** Active or athletic individuals should follow the same precautions as the rest of the population, as they are at the same risk of contracting the disease as any other human being.

- Many of us had childhoods filled with physical activity. We played hula hoop, jump rope, hopscotch, and hide and seek, as well as other games that we still remember and that remind us a time with no pandemics but that will inexorably never return. Faced with the current scenario, why not take advantage of this time with your children; reiterating: make the most of this time with your children, which was something many of us could not imagine was possible a few weeks ago!!! Drag them out of bed or off the sofa; encourage them to give WhatsApp, games, and Netflix a break. Go play!!!

**Sixth point:***Return to the past* . Encourage your children to be less sedentary. According to the World Health Organization, it is ideal that adults dedicate 150 minutes to exercise weekly and that younger individuals exercise for at least 300 minutes weekly.^2^ In short, integration of the family promotes the family’s own health.

- Not all physical activity is exercise, but all exercise is a form of physical activity. There are countless examples, sweeping, ironing, cleaning and/or vacuuming, gardening, working in the yard, going up and down the stairs (with precaution if the stairs in an apartment building are being used by other residents at the same time), and others.^2^

**Seventh point:** Intensify physical activity at home on a daily-to-day basis. You and your family will have a more active, healthier, and more productive lifestyle.

- Sexual activity is a form of physical exercise/activity. For many, it is the most pleasurable one. In times of isolation-coronavirus, this is a general concern. We certainly do not have scientific data, but logic leads us to think that distancing should come before desire, that common sense should be more prevalent than the pleasure principle.

**Eighth point:** Unfortunately, it is recommended that sexual activity involving hugging, touching, kissing, and penetration be avoided (in the scenario of occasional sexual activity, of course). Masturbation, the use of vibrators and erotic toys is not contraindicated. If objects are to be shared, it seems to me that they should be washed with water and soap and/or 70% alcohol gel (this is merely an intuitive deduction, given that there are no studies testing hypotheses on this subject).

- Readers of the Archives, regarding this theme, it would certainly be possible to list several other points, but it seems to me that at the end of these indications what is most important is to emphasize that physical inactivity in a treacherous enemy. Its effects typically do not manifest in an acute manner as is the case with SARS-COV-2, whose effects need to be combated with emergency health measures based on the best scientific evidence. In turn, by negatively affecting the cardiovascular, respiratory, metabolic, muscular, and immune systems, physical inactivity is also very harmful. In conclusion, the previous eight points were written in order to provoke reflection that at this moment we must live within the confines imposed by this new global order that is restrictive, but not limiting.
